# The Transcriptomic Toolbox: Resources for Interpreting Large Gene Expression Data within a Precision Medicine Context for Metabolic Disease Atherosclerosis

**DOI:** 10.3390/jpm9020021

**Published:** 2019-04-29

**Authors:** Caralina Marín de Evsikova, Isaac D. Raplee, John Lockhart, Gilberto Jaimes, Alexei V. Evsikov

**Affiliations:** 1 Department of Molecular Medicine, Morsani College of Medicine, University of South Florida, Tampa, FL 33612, USA; iraplee@health.usf.edu (I.D.R.); jlockhar@mail.usf.edu (J.L.); gjaimes@health.usf.edu (G.J.); 2 Epigenetics & Functional Genomics Laboratories, Department of Research and Development, Bay Pines Veteran Administration Healthcare System, Bay Pines, FL 33744, USA; aevsikov@health.usf.edu

**Keywords:** atherosclerosis, coronary aortic disease, gene set enrichment analysis, heart disease, metabolic disease, transcriptomics, pathway enrichment analysis, RNA-seq analysis, secondary gene expression analysis

## Abstract

As one of the most widespread metabolic diseases, atherosclerosis affects nearly everyone as they age; arteries gradually narrow from plaque accumulation over time reducing oxygenated blood flow to central and periphery causing heart disease, stroke, kidney problems, and even pulmonary disease. Personalized medicine promises to bring treatments based on individual genome sequencing that precisely target the molecular pathways underlying atherosclerosis and its symptoms, but to date only a few genotypes have been identified. A promising alternative to this genetic approach is the identification of pathways altered in atherosclerosis by transcriptome analysis of atherosclerotic tissues to target specific aspects of disease. Transcriptomics is a potentially useful tool for both diagnostics and discovery science, exposing novel cellular and molecular mechanisms in clinical and translational models, and depending on experimental design to identify and test novel therapeutics. The cost and time required for transcriptome analysis has been greatly reduced by the development of next generation sequencing. The goal of this resource article is to provide background and a guide to appropriate technologies and downstream analyses in transcriptomics experiments generating ever-increasing amounts of gene expression data.

## 1. Introduction

Often starting in adolescence [1], atherosclerosis is an initially asymptomatic ‘silent’ disease, as the artery slowly narrows from the gradual accumulation of plaques, which consist of fat, cholesterol and calcium, and often harbor bacteria [2]. As oxygenated blood flow decreases over time, symptoms emerge at middle age, and atherosclerosis disease progression spurs stroke, peripheral artery disease, kidney problems, heart disease and coronary artery disease [2]. Atherosclerosis is very costly [3]; for example, in the United States it accounts for 1.3% of hospital stays and costs $9 billion per year with all atherosclerosis-related morbidities accounting for $43.5 billion of total hospital costs per year [4,5]. While etiology is complex, inflammation is currently proposed to be one of the initial triggers for atherosclerosis [6]. Diagnosis focuses on the detection of severe arterial narrowing using physical examination, electrocardiograms, and exercise-induced stress testing, but not directly the underlying atherosclerosis disease itself [7]. Treatment of established disease typically focuses on alleviating symptoms arising from pathophysiology, starting with modifying lifestyle risk factors, such as diet restrictions and exercise to increase arterial circulation, decrease obesity and blood pressure, and cessation of smoking to prevent deposits of plaque, combined with pharmaceuticals to lower cholesterol, such as statins, blood pressure medication such as diuretics and β-blockers, or decrease clotting, such as aspirin [8,9].

Treatments that target molecular mechanisms underlying the physiological changes, in addition to treating symptoms arising from pathophysiology, are a central promise of personalized medicine—indeed, in theory, genomic data can reveal specific disease-associated associated genotypes to optimize the treatment plan [10]. In reality, only a few exact genotypes have been identified, such as homozygous deletion of angiotensin-converting enzyme I (*ACE*) [11], aryl hydrocarbon receptor (*AHR*) polymorphisms [12], and in many different studies, multiple alleles of apolipoprotein E (*APOE*) [13,14]. An alternative to the clinical genetics and population genetics approach is the identification of altered pathways via transcriptome analysis of atherosclerotic tissues. Transcriptome analysis allows for the detection differentially expressed genes in atherosclerotic tissue that may drive its pathogenesis. The variety of technologies available to researchers makes choosing the most appropriate platform to address and resolve specific scientific problems (or hypothesi) using transcriptome analysis a daunting task. While some researchers believe microarrays are the most reliable due to their maturity, others embrace next-generation sequencing (NGS) as the superior method because it is the current vanguard of molecular technology. Assumptions in data analysis can skew and obviate the resulting data interpretation of gene expression if the hypothesis and, most importantly, the experimental design do not mitigate the shortcomings of each platform. After gene expression has been measured, the researcher must also choose from numerous software programs and analyze expression data. Therefore, the goal of this resource article is to provide explanations of the origins, strengths, and limitations of the wide-ranging transcriptomic technologies as it pertains to gene expression analysis as guide to resources available to interpreting transcriptome experiments within the context of atherosclerosis research. As the application of transcriptomics to the field of atherosclerosis as precision medicine is in its infancy, this knowledge will assist researchers in choosing an appropriate sequencing technology and bioinformatics analysis methods to address biomedical problems and questions in atherosclerosis research addressed by experimental studies of gene expression.

## 2. Materials and Methods Used in Transcriptomic Studies

**Figure 1:**
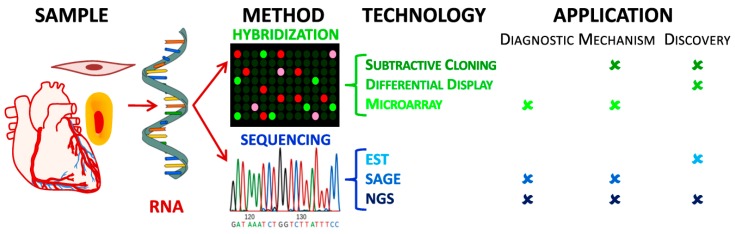
Transcriptomics workflow diagram highlighting the steps to process tissue, cell, or biopsy sample for RNA and choosing gene expression technology platform depending upon the specific application as an investigative tool for discovery science, disease diagnosis, or molecular mechanism. EST: expressed sequence tag; SAGE: serial analysis of gene expression; NGS: next-generation sequencing.

Transcriptome analysis creates a detailed molecular synopsis of cellular physiology by elucidating the mRNA available for translation and/or the abundance of other types of transcripts, such as noncoding RNAs or microRNAs. Techniques used in transcriptome analysis belong to two broad classes; hybridization-based or sequencing-based (Figure 1). The time and cost of transcriptome analysis has been greatly reduced by the development of microarrays and, more recently, NGS, when compared to older gene expression analysis technologies, such as expressed sequence tag (EST) libraries, or serial analysis of gene expression (SAGE). Given the variety of factors affecting atherosclerosis and the multiple pathways involved, transcriptomics is a useful tool for diagnostics, discovery science, and pinpointing molecular mechanisms in both clinical and translational models of disease. Transcriptomics provides a way to identify treatments and therapeutics with the greatest potential to affect the cellular and molecular mechanisms underlying atherosclerotic disease, in addition to novel therapeutic approaches to alleviate symptomology (Figure 1).

### 2.1. Origins of Transcriptomics: Gene Expression in the Evolution of Hybridization Techniques and Sequencing for RNA Identification

#### 2.1.1. Classic Hybridization-Based Technologies: Subtractive Cloning & Differential Display

Subtractive cloning is an inexpensive and common technique in individual biomedical and clinical laboratories to analyze gene expression using readily available molecular biology resources. It is a hybridization technique used to detect genes specific to a cell or tissue using a subtraction protocol to remove all common sequences between control and experimental cDNA libraries (i.e., cell type, drug treatment, disease condition etc.) yielding a specific library representing differentially expressed genes. Hybridization of cDNA may cause bias for small fragments of cDNA that hybridize faster than long sequences, but is resolved by PCR amplification [15]. These enriched libraries can be used in conjunction with other transcriptomics techniques, such as microarray or NGS (2.1). Likewise, the other common routine hybridization technique, Differential Display, is a PCR-based method that also detects and measures differential gene expression without using specific primers, making it a robust, inexpensive discovery tool [16]. Current innovations incorporate the use of fluorescent labels with automation to yield high throughput analyses [17,18]. These techniques have been applied successfully to understand molecular and cellular pathways involved in stem cell differentiation by identifying the expressed genes causing lineage commitment into megakaryocytes, erythrocytes, and granulocytes, which play different roles in atherosclerosis disease developmental and disease progression [19].

#### 2.1.2. Modern Hybridization-Based Technologies: Microarrays

**Figure 2:**
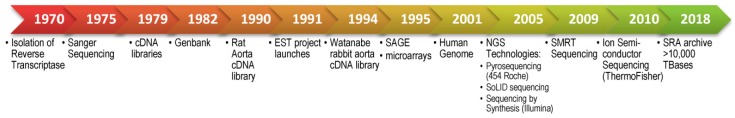
Timeline of the introduction of prominent technologies for gene expression measurement and bioinformatics analysis since the discovery of reverse transcriptase, an enzyme indispensable for any RNA sequencing study. Some of the seminal papers in atherosclerosis research discussed in the text are shown in this timeline as well. Timeline is not to scale. SMRT: single molecule real time.

**Figure 3:**
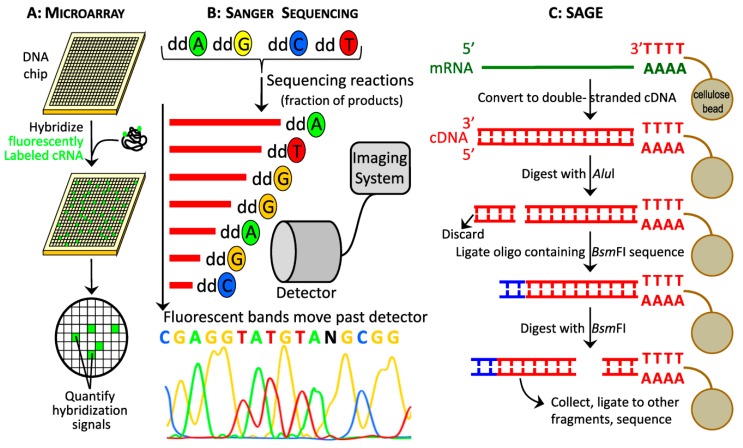
Older technologies used in gene expression studies. (**A**) In microarray experiments, labeled cRNA are used to measure the gene expression level by hybridization to cDNAs on glass slides representing known genes. The intensities are measured, normalized, and analyzed by computer software to compare experimental treatments or conditions. (**B**) Sanger sequencing was the original method of measuring DNA nucleotide sequence based on chain-dye termination and the first technology for sequencing of expressed genes. (**C**) Steps in producing concatenated short tags for subsequent sequencing in SAGE method.

In 1995, cDNA microarrays superseded the method of Differential Hybridization, introducing the use of miniature spotted DNA probes and fluorescent labeling of samples, reducing the redundancies after hybridization (Figure 2). Pools of known cDNAs (spots) in indexed locations on glass slides represent known genes (Figure 3A). Total sample mRNA is reverse transcribed, cRNA amplified by in vitro transcription, and then hybridized to microarray slide. The intensities of the spots produced are then recorded and analyzed by computer software to determine the expression level of a gene (Figure 3A) [20]. One advantage of cDNA microarrays over EST or SAGE sequencing techniques (Section 2.1.3) is the ability to analyze gene expression differences under various experimental conditions concurrently by using different fluorophores during the cRNA transcription (Figure 1 and Figure 3A). Microarray analysis requires substantially less poly(A) RNA (0.5–2.0 µg) compared to Subtractive Cloning, Differential Display or EST libraries methods (Figure 1), albeit microarray limitations are the quality, specificity, and signal discrepancy of the probes on the array. After introduction, microarray analysis became common and made labor-intensive EST and SAGE libraries essentially obsolete, despite that this method detects only previously discovered transcripts (e.g., from EST libraries) and its inherent inability to discover novel genes, alleles, or splice variants (Figure 1) [21]. With its high throughput method requiring low manual labor, low amount of starting RNA, and streamlined bioinformatics processing, microarrays provide an attractive alternative to sequencing for transcriptome analysis. Examples of microarray technology use in atherosclerosis research are studies on the impact of cellular senescence on gene expression patterns in vascular smooth muscle cells (VSMCs) [22], and identification of PPAR signaling pathways in animal models of atherosclerosis [23].

#### 2.1.3. First Generation Sequencing: Sanger Sequencing, cDNA libraries, EST and SAGE

Sanger sequencing is the keystone invention for modern methods, such as NGS, to sequence for expressed genes in transcriptomic studies. Sanger sequencing is the “first-generation” method of determining DNA nucleotide sequence based on the chain-termination idea developed in 1975 [24] (Figure 2). Modern modification of this classic method is based on in vitro DNA elongation of target template, which is interrupted by labelled di-deoxynucleotides (ddNTPs) to halt DNA strand synthesis for sorting and fluorescence detection (Figure 3B) [20]. Expressed genes are identified using various methods to harvest RNA to make and use cDNA for expression studies, including subtractive cloning, EST, SAGE, differential display analysis, and microarray analysis (Figure 1 and Figure 3). Once identified, gene interactions with other genes can be pursued experimentally [20]. In the late 1970s, cDNA libraries [25] became popular for gene discovery and expression analysis, as the library clones were stable, reproducible, and recoverable representations of mRNAs isolated from distinct organs and species, although they were not embraced in the field of atherosclerosis for over 10 years until the early 1990s with sequencing of rat and rabbit aorta cDNA libraries (Figure 2) [26,27]. Meaningful data are generated with high throughput preparation of either normalized or non-normalized cDNA libraries [28].

Expressed sequence tags are derived from cDNA libraries by random sampling, followed by arraying and single-pass sequencing of the sampled clones; array replicas may be stored frozen for future use. ESTs allow for *de novo* gene discovery [26,27], and large-scale prediction of gene products and function (Figure 1) [29,30]. Expressed sequence tags analysis was used to identify genes overexpressed in the mouse model of atherosclerosis [16], and high-quality EST data, including heart and atherosclerosis, are available in Unigene and ENSEMBL. The next development in transcriptomics was SAGE in 1995 (Figure 3C). SAGE constructs cDNA libraries in a similar fashion to ESTs, but the end product are concatenated short tags used to identify genes (Figure 3C). One advantage of the SAGE method is the high-throughput sequencing capability, although the bioinformatics tools required to analyze the libraries are highly specialized. SAGE analysis can be successfully used for *de novo* expression profiling, but the short length of the SAGE tag can impair differentiating between highly homologous genes. In atherosclerosis research, SAGE was successfully used to study in vitro human endothelial cells response to atherogenic stimulus (conditioned medium of oxidized-LDL-stimulated monocytes) [31], and to identify biomarkers of atherosclerosis in circulating human monocytes [32].

### 2.2. Next-Generation Sequencing and Deep Transcriptome Analysis

Second generation sequencing techniques emerged in 2005 (Figure 2), and equipment fundamentally differs from first generation sequencers because multiple different DNA molecules are sequenced concurrently. As a result, tens of thousands to hundreds of millions of individual sequencing reads are produced with each run. Different principles underlying sequencing and detection, and different chemistries behind various platforms lead to large differences in read length, base call accuracy, and total number of output reads. The largest obstacle for second generation sequencers is obtaining read length to read quality ratios comparable to Sanger sequencing, with most platforms producing average reads with less than 300 bases. In addition, the samples are sequenced in a stop-read-start manner that leads to lengthy processing times, with some platforms requiring over a week for a single run to complete. To make these platforms economical, the number of reads per run has been increased through the introduction of larger machines, such as the Illumina HiSeq series, or denser chips, in the case of Ion Torrent. However, the larger sequencers have a substantially higher price and require processing at full capacity to benefit from the increased throughput and, consequently, are not typically found in individual laboratories or small research consortia. There are smaller platforms available from Illumina, 454 Roche, and Ion Torrent that produce longer length reads than the larger sequencers, thereby suit the needs of small research consortia and well-funded laboratories [33].

#### 2.2.1. Basic Principles of NGS Sequencing

All second generation sequencing platforms require modification and amplification of sample DNA. Samples are fragmented and adapters are annealed to the ends. For platforms that use emulsion PCR (emPCR) to amplify the samples, the adapters allow the fragments to bind to complementary bases on the emulsion beads. SOLiD sequencing further modifies the fragments after amplification by adding regions that allow the fragments to covalently bond with the sequencer slide. The Illumina platform uses a bridge PCR to amplify the samples, which have been modified with adapters to the base pair with oligonucleotides embedded on the sequencer slide.

**Figure 4:**
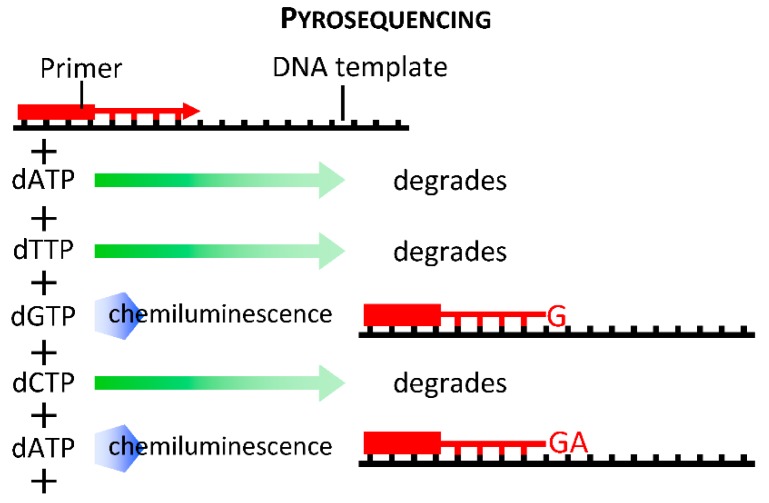
Principle of pyrosequencing.

Each platform also employs a different method for generating the base calls for each sample, but only Ion Torrent does not use a light-based recording method. The base calls are reported by pyrosequencing (Figure 4) in 454 Roche platforms, and by fluorescent tag cleavage in Illumina and SOLiD platforms. The Illumina platform produces forward and reverse reads from each DNA fragment and SOLiD identifies each fragment’s bases twice, thereby increasing accuracy. Ion Torrent uses a microchip with pH meters incorporated into each well to detect the release of an H^+^ ion with each base incorporated.

Extension of fragments occurs during sequential “flooding” of the sequencing reaction chamber with solutions containing specific nucleotides. Illumina differs from other platforms by using a reaction mixture containing all 4 nucleotides. The Illumina nucleotides are modified with a fluorescent group plus a terminator to prevent introduction of additional bases in the cycle. The fluorescence is recorded and its tag cleaved before flooding the sequencer with the nucleotide-containing reaction mixture again. In pyrosequencing (Figure 4), the nucleotides have a modified pyrophosphate group that is cleaved after addition. SOLiD sequencing uses di-base oligonucleotides with a 3-base extended region and a fluorescent tag. An (n+1)-long primer is added after each round of synthesis which, after 5 repetitions, emits two base signals for each incorporated nucleotide. Nucleotides in Ion Torrent sequencers are added in alternating “floods” of A, T, C, and G. As each base is paired to the fragment, an H^+^ ion is released and detected by the sequencer microchip.

#### 2.2.2. Development of Single-Cell RNA Sequencing Strategies

The recent ability to interrogate the transcriptome of individual cells using second generation sequencers has revealed heterogeneity in gene expression of individual cells within a population. As the name implies, single-cell RNA sequencing (scRNA-seq) relies on the isolation and amplification of transcriptomes from individual cells, and many different isolation and amplification strategies have been developed, such as Cel-seq2 [34], Smart-seq2 [35] and Drop-seq [36]. Isolation of individual cells is accomplished by using microfluidic capture chips (Cel-seq2), fluorescence activated cell sorting (Smart-seq2), or droplet emulsion (Drop-seq). Most scRNA-seq protocols, excluding Smart-seq, incorporate cell-specific barcodes during the reverse transcription reaction that allows for large-scale multiplexing. Smart-seq, in contrast to other scRNA-seq methods, generates full length cDNA and can more accurately differentiate between splice variants. A side-by-side comparison of these scRNA-seq strategies found that Drop-seq was the most cost-effective method, whereas Smart-seq was the most accurate [37]. Analyzed cells may be clustered based on expression levels of selected genes either to detect changes in cell populations or within a population induced by a disease. This strategy to separate and sequence by cell type was recently used to analyze normal and atherosclerotic aortas from mice and detected a previously unreported population of macrophages that expressed high levels of triggering receptor expressed in myeloid cells 2 (Trem2) gene in diseased aortas, including atherosclerosis [38].

#### 2.2.3. Strengths & Caveats for Transcriptome Analysis

Next generation sequencers are powerful tools, but they are not without flaws and errors that can arise at any step of the sequencing process. Firstly, errors may be introduced by polymerase during the amplification of sample cDNA, and research indicates that this may be the primary source of errors in second generation sequencing data [39]. Secondly, errors originate from the chemistry used by the various platforms, and often manifest in nucleotide substitutions, insertions, or deletions [33]. The error rates of second generation sequencers are principally increased in homopolymeric regions caused by the incorporation of multiple bases in a single cycle. AT-enriched regions and genomes cause increased error rates in next generation sequencers, possibly from PCR artifacts and nonrandom fragmentation of sample DNA [40]. Errors due to AT-richness are most pronounced in the Ion Torrent platforms [41]. Furthermore, when utilizing single-cell sequencing strategies, comparison between samples can be greatly impaired by poor matching of samples, the stages of disease progression, and the variability between individuals can compound the inherent heterogeneity that is present when comparing individual cells. While the ability to determine the response and contribution of individual cell types to disease progression is important, more samples are necessary to identify and distinguish between inter-individual and intra-individual variations.

For next-generation RNAseq analysis, the most important parameters to consider in experimental design in order to substantially increase the quality of downstream analysis are: the number of biological replicates, the depth of sequencing (i.e., number of reads produced for each sample), read length, single-end vs. pair-end sequencing (i.e., each sequenced DNA molecule is represented by a single strand read vs. two reads from each strand), and RNA extraction. Under budgetary constraints, tradeoffs between sequencing depth and the amount of biological replicates are often made. As consistently reported, the requisite number of biological replicates (*n* = 3–4) is more critical for robust, reliable, and replicable analysis than sequencing depth [42,43,44,45]. As technologies improve, sequence lengths increase. For differential expression, little difference is seen if the length is >25 bps, in either single-end or pair-end sequencing. However, for greater accuracy in transcript identification and splice junction detection, reads should be pair-end and ≥100 bp [46]. The RNA extraction method impacts the ratio of RNAs present during sequencing, and a specific strategy should be chosen with the biological or biomedical question of interest in mind. For example, total RNA extraction is useful in capturing unique transcriptome features, such as noncoding RNA. However, ribosomal RNA (rRNA) comprises >90% of total RNA and should be depleted if noncoding, non-ribosomal RNA is to be assessed. Current techniques cannot completely remove rRNA, and ~2%–35% residual remains in the sample. Therefore, greater sequencing depth should be considered when using ribosomal depletion methods to counter the abundance of rRNA and improve detection of other transcripts. In eukaryotic organisms, if only protein coding genes are of interest, poly(A) selection yields greater accuracy of transcript quantification [47]. These issues are particularly critical for clinical samples from patients, which are routinely processed as formalin-fixed, paraffin-embedded (FFPE) samples, which adversely impact the quality of RNA and subsequent alignment to pseudogenes [48]. Fortuitously, side-by-side comparison of FFPE and flash-frozen samples shows a great degree of concordance (e.g., *r*^2^ in the range of 0.90–0.97 in recent studies [49,50]), proving RNAseq is a viable tool for gene quantification in clinical settings. Controls, depending upon availability, need to be non-diseased tissue, either of the same patient origin or from another individual without the disease [51]. In addition, given atherosclerosis is a common disease, patients are from genetically diverse, heterogeneous populations with variable symptomology, which requires more samples to detect meaningful changes in the transcriptome truly reflecting disease process. However, in other diseases, such as breast cancer, as few as *n* = 9–10 patient samples (plus samples of healthy controls), have been ample to detect specific alleles and molecular pathways [51].

Despite the errors that may occur when using second generation sequencers, several advantages over the original transcriptome technologies, such as Sanger sequencing, EST and SAGE (Figure 1, Section 2.1), warrant their use experimentally and clinically. First of all, second generation sequencers offer orders of magnitude deeper coverage of sample RNA than achieved by Sanger sequencing, via EST libraries, yielding overall faster discovery and more accurate analysis of an entire transcriptome. Also, the length and quality of sequence produced by second generation sequencers are much better than the fragments produced in SAGE, which improves transcriptome accuracy. While EST sequencing typically produced fragments of at least 500 bp, most second-generation sequencing produces shorter read lengths, albeit, read length from second generation sequencers can be increased at the expense of read depth. Next generation sequencers have advantages over microarrays because essentially all expressed transcripts and their variants can be detected, without restriction to the probes present on the microarray chip or beads [52], plus the ability to barcode different samples, or conditions, within a single sequencing procedure permits multiplexing of samples.

### 2.3. Third Generation Sequencing

The latest generation of sequencers is distinguished from first and second generations by eliminating sample amplification. Bypassing sample amplification reduces sample preparation time and eliminates signal mismatch and distortion errors introduced during amplification. In addition, these single-molecule sequencers produce extremely long reads, surpassing the lengths achieved by Sanger sequencing. The Pacific Biosciences Single Molecule Real Time (SMRT) sequencer utilizes pyrosequencing (Figure 4) in polymerase-embedded plates, which lower the signal-to-noise ratio to detect real-time signal processing of fluorophore cleavage. The use of pyrophosphate-labeled nucleotides in polymerase-containing plates to extend DNA at near its natural speed facilitates processivity and output length of sequencing read. Another third generation sequencing platform available now is nanopore sequencing (MinION, Oxford Nanopore Technologies, Oxford Science Park, Oxford, UK). This technology utilizes electrophoresis of DNA molecules via nanopores (5–8 nm diameter); as the DNA molecules squeeze through the pore, each nucleotide (A, T, G and C) produces a unique electromagnetic signature is detected. Similar to SMRT, nanopore sequencing can produce very long reads, up to 880 kb in a recent report [53].

#### Strengths & Caveats for Transcriptome Analysis

The Nanopore and SMRT sequencer both have ~10–15% error rate, distributed evenly over the length of the read [53]. Fortunately, the lack of location bias in SMRT and Nanopore reads provide sufficient coverage to extrapolate highly accurate consensus sequences. Third generation sequencers are not yet ubiquitous, but they promise several advantages over previous generation sequencers. The lack of sample amplification allows for quicker, cheaper analysis and avoids the polymerase errors caused by amplification. The long reads generated by third generation sequencers allow for more accurate assembly of large contiguous sequences, such as whole chromosomes, complete sequencing of whole genes in a single read [54], and identification of novel transcript isoforms. These platforms are excellent for whole-genome and whole-transcriptome assemblies [55,56], including complex genomes such as gorilla [57] and human [53]. However, at this time, third generation sequencers are at a disadvantage for use in transcriptome analysis for quantification of expression due to the relatively low number (e.g., ~50,000 for RSII sequencer) of output reads generated with each run comparing to, e.g., Illumina sequencers (current typical low-end is 20,000,000+ reads per sample). The long reads greatly improve *de novo* assembly and transcriptome analysis for gene isoform identification, and the emerging technology in the field of metagenomics, which may be important for investigating the role of microorganisms in the onset of atherosclerosis. Longer reads are also useful when assembling genomes that include large stretches of repetitive regions. These technologies are recommended for whole genome assembly and splice variant detection, albeit given the error rate currently not recommended for transcript quantification.

## 3. Results of Transcriptome Analysis: Unbiased Data Mining to Find a Needle in a Haystack

### 3.1. Differential Expression Analysis

**Figure 5:**
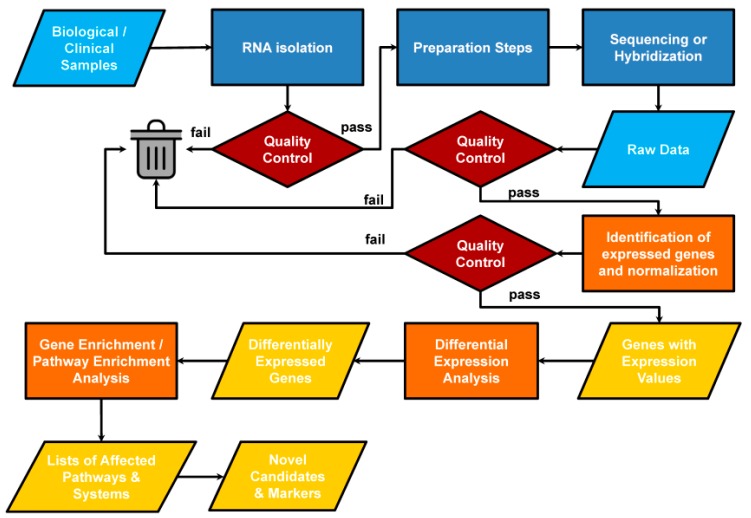
Generalized pipeline for a high-throughput microarray or RNA-seq transcriptomics study.

In most cases, comparison of one or more conditions will result in a ranked list of transcripts with either relative or absolute levels of expression. The typical approaches include: (1) raw data collection (processing of image files to collect intensities for individual probes on microarrays, counts of number of reads per transcript for RNAseq data, etc.); (2) data normalization, often followed by transformation [58]; (3) statistical analysis to identify transcripts whose expression differences between conditions are significant, and most importantly, (4) downstream analysis (Figure 5).

Microarrays of any platform are substantially more rapid to process using the manufacturers’ software suites, such as Affymetrix’s Expression Console and Transcriptome Analysis Console, or Illumina’s GenomeStudio. Alternative open-source, peer-reviewed, and publicly available software for microarray analyses using the R programming language, such as affy [59], lumi [60] and limma [61], are available as installation packages from the Bioconductor portal [62]. For next-generation RNAseq analysis, the most important parameters to consider in experimental design that substantially increases the quality of downstream analysis are depth of sequencing (i.e., number of reads produced for each sample, also referred to as “coverage”), read length, and single-end vs. paired-end sequencing. These parameters vary based on the goal of the biological or clinical experiment. For example, comparison of expression between samples requires far less read depth than the identification of novel transcripts or splice variants. Journals that publish RNAseq studies sometimes have their own requirements for read depth. Furthermore, the length of sequencing reads varies depending on experimental design, with longer reads typically being used in novel transcript identification or *de novo* assembly generation [56]. Sequence read lengths as low as 75 bases are sufficient for differential expression analysis [43]. Finally, paired-end sequencing from both ends of a single mRNA fragment facilitates identifying splice variants and alignment [46].

Once the sequence is obtained from the raw signals, the quality of the output must be assessed, based on sequence read lengths and processing direction (single-end vs. paired-end sequencing) with either FastQC [63] or NGSQC [64]. These tools will provide GC content, overrepresented reads, PCR artifacts, and sequence quality to detect potential PCR bias or DNA contamination. It is normal for sequence quality to weaken at 3′ end and software programs, such as Trimmomatic [65] or FastQ trimmer [66], can remove these low-quality 3′ ends. Alignment is a critical step in RNA sequencing analysis because raw sequence reads must be mapped precisely to an annotated reference genome or transcriptome for the species. While it is possible to analyze RNAseq data without a reference, e.g., by using Trinity software [67], most clinical and translational models of atherosclerosis have assembled genomes available. The most common software platforms to align RNA sequence to a reference genome are TopHat [68], HiSAT [69], and STAR [70]. These platforms differ with respect to speed, memory usage, and their algorithms for handling pseudogenes [48], base and splice junction alignment precision, with HiSAT and STAR optimized to process large datasets (>10^8^ reads), whereas TopHat is designed for smaller datasets (<2 × 10^7^ reads).

Measurement of transcript expression in RNAseq data is based on quantifying raw counts at each genetic locus along the chromosomes using an assembled genome with programs such as HTSeq-count [71] or featureCounts [72]. This approach uses a GFF (Generic Feature Format) or GTF (General Transfer Format) file that contains gene coordinates, identifiers, and descriptions in a strict predefined format [73]. All the reads that map within the genomic coordinates of a given feature (e.g., gene, exon) contribute to the count number of this feature. The counts from the RNAseq data are corrected for sequencing depth, and often for length of gene transcripts because smaller datasets will have fewer count numbers, with the consequence that longer transcripts will have a higher representation among raw RNAseq reads. The majority of normalization methods report the amount of transcript expression as reads per kilobase of exon per million reads (RPKM), fragments per kilobase of exon per million of reads (FPKM), transcripts per million (TPM), or counts per million (CPM) [48,74,75,76].

### 3.2. Categorical Enrichment Analysis: An Overview of Biological Ontologies 

**Figure 6:**
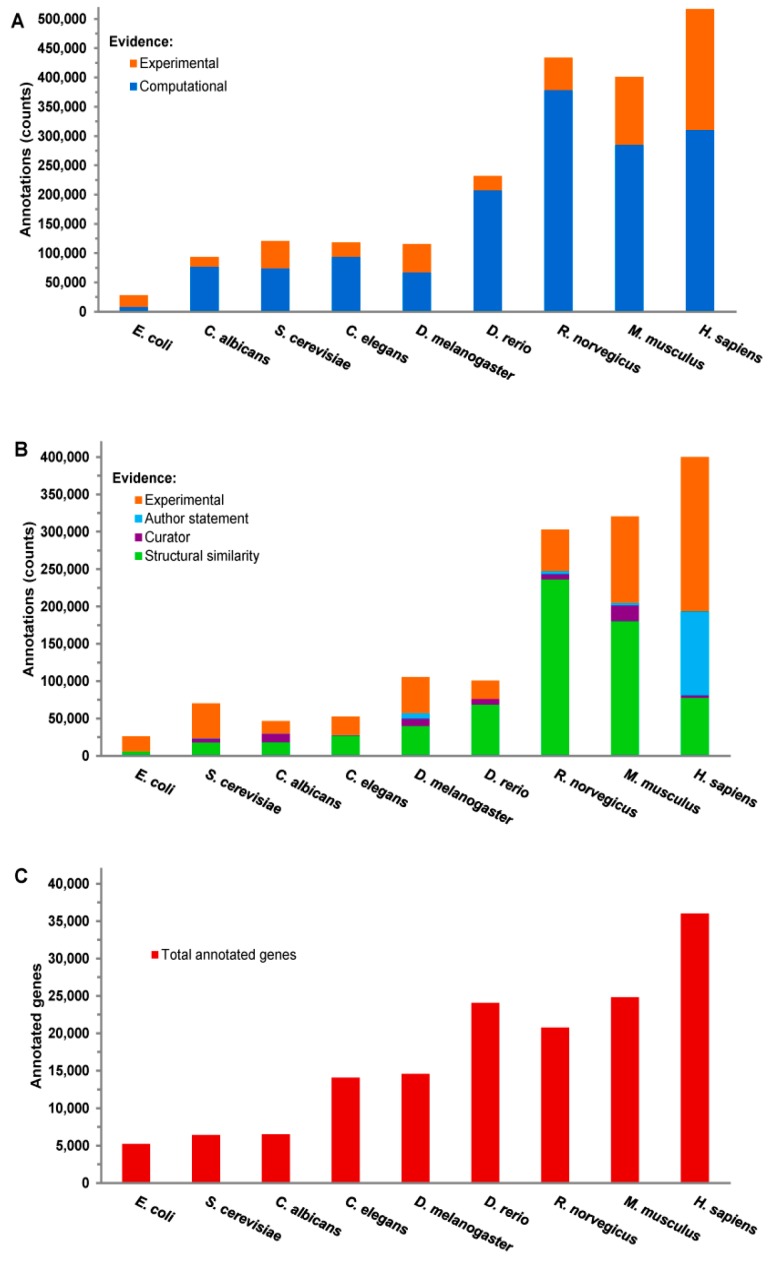
(**A**) Gene annotations in Gene Ontology (GO) across species based on type of evidence supporting gene annotation. (**B**) Breakdown of gene annotations based on most frequently used evidence categories (Biological Process, Cellular Component, and Molecular Function categories combined). (**C**) Number of genes annotated with at least one GO term in the species.

Description of gene functions in scientific literature can vary significantly among authors, even if all of them are describing the same phenomenon. Consequently, unbiased grouping of genes by functional similarities may become a daunting endeavor. To facilitate the task of classifying the universe of genes, the methods of formal ontology were applied to create the first controlled vocabulary to standardize gene descriptions across species and disciplines. The resulting Gene Ontology (GO), and GO Consortium were formed in 1998 to create a framework for standardizing gene products description [77]. Since its inception, GO was used to annotate millions of genes, with over 1,350,000 annotations for *H. sapiens*, *R. norvegicus*, and *M. musculus* genes alone [78] (Figure 6A). The highest-level annotations for genes in GO is a “trinity” of Molecular Function, Cellular Component, and Biological Process hierarchies. Currently, GO uses 29,623 “Biological Process”, 11,139 “Molecular Function”, and 4,189 “Cellular Component” terms, and strict rules to describe evidence linking a gene to a term (from relatively vague “Inferred from Sequence or structural Similarity” to strong “Inferred from Experiment”), to annotate genes across the tree of life (Figure 6B); taking into account the total number of annotated genes in species (Figure 6C), currently average number of GO annotations ranges from 5 for *E. coli* to 21 for *R. norvegicus*.

**Figure 7:**
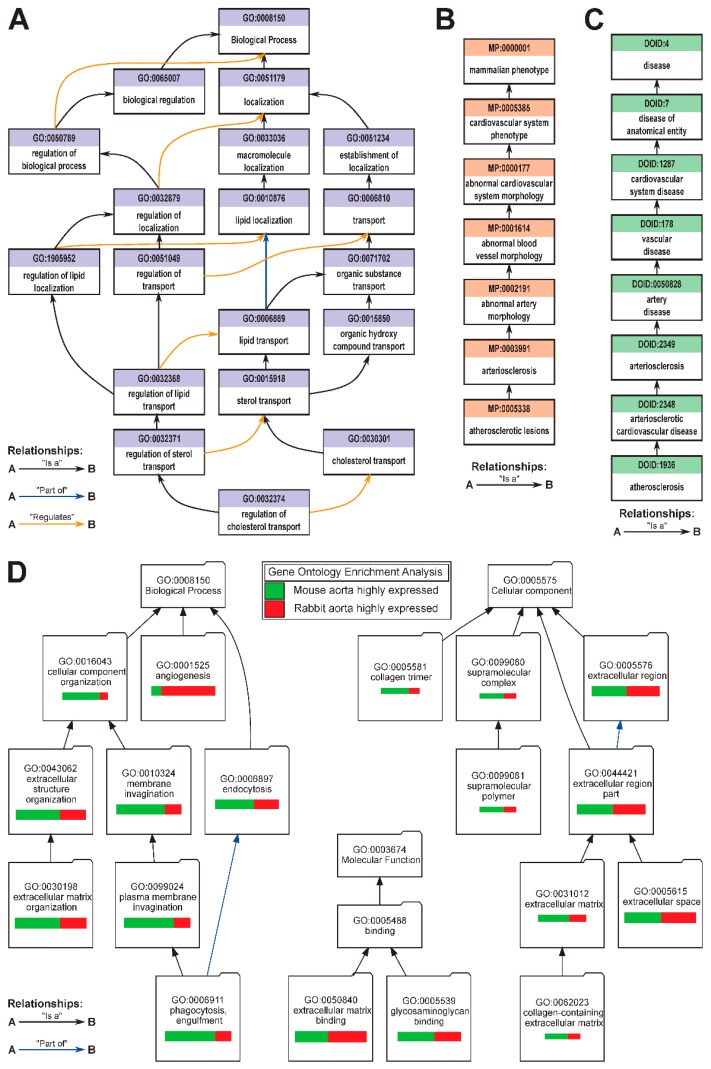
Examples of ontology structure and the power of ontological analysis. Gene Ontology (GO) (**A**), Mammalian Phenotype Ontology (MP) (**B**), and Human Disease Ontology (**C**) terms related to atherosclerosis. (**D**) Bioinformatics analysis result for VisuaL Annotated Display (VLAD) illustrating the statistically significant GO categories overrepresented among the 100 highest-expressed genes in atherosclerotic aortas of mouse and rabbit translational models. The width of the color bar represents the relative “strength” of a particular GO pathway representation among the highest-expressed genes and exemplifies similarities and differences between models, green bar = mouse, red bar = rabbit.

GO is organized as a graph, with individual terms being nodes, and relationships between terms being edges. For example, one of the GO annotations of an “atherosclerosis gene” *Apoe* is “regulation of cholesterol transport (GO:0032374)”; the relationship of this term to higher-level terms is shown in Figure 7A. Currently, there are eight types of relationships between terms, and the *“is_a”* relationship gives this ontology a loose hierarchy, with more general terms being “parent” to more specific “child” terms [77]; other common relationships are *“part_of”* and *“regulates”* (Figure 7A). Curation remains an ongoing process, including the field of cardiovascular disease and atherosclerosis [79], and new annotations, and new GO terms are added frequently as scientific and specific knowledge expands. The dynamic nature of GO catalyzes new discoveries to be readily integrated into the existing ontology, while older annotations are updated with new information as it becomes available. Following the success of GO, other ontologies began to emerge to formalize biological and biomedical knowledge to assist in large-scale data analysis and discovery of new treatment avenues. Relevant examples (Table 1) include Mammalian Phenotype Ontology [80] and Human Disease Ontology [81,82], both used to formalize descriptions of normal and disease phenotypes, in our example specific for atherosclerosis (Figure 7B,C). Another example Ontology, Protein Ontology, describes evolutionary relation, isoforms, and complexes of proteins [83,84]. All these, and many other ontologies collectively form an Open Biological and Biomedical Ontology (OBO) Foundry and share common goals to facilitate curation, management, distribution, and analysis of data [85].

## 4. Discussion: Using Ontologies & Pathway Analysis for Precision Medicine

Unlike DNA sequencing focusing on genome, RNA sequencing produces the snapshot of the full transcriptome supporting its potential capability to fulfill precision medicine to classify patients at both molecular and cellular levels when used in conjunction with programs for ontologies and pathway analysis. Development of RNA sequencing pipelines is important for implementation of transcriptomics as precision medicine [94], which can be used successfully to classify patient or model attributes and predict therapeutic response and ultimate outcomes [48].

The first step of ontological analysis of genes is the annotation of the gene, assuming it has not been previously annotated. Once all the gene annotations have been collected they are grouped by category, and these categories are analyzed for enrichment or depletion against a “universe set” of all the genes of an organism. The number of annotations to a distinct ontological term in a list of genes, for example, a list of downregulated genes in atherosclerotic vs. normal aorta is compared to the number of annotations to this term among genes in the universe set (i.e., all genes in the genome) to identify if the occurrence of this term in the experimental results is higher or lower than expected from a random sampling of the universe set. This analysis facilitates discovery of common biological themes, based on ontologies, within the lists of genes. Multiple tools exist for determining pathway enrichment; among preferred tools in our laboratory is the VisuaL Annotation Display (VLAD) [85], which allows to define the “universe set” (i.e., the background list of genes), rather than just GO. An example of VLAD analysis (Figure 7D) compares the overrepresented GO terms among the 100 highest-expressed genes in aortas of *Apoe*^-/-^ mice fed Western diet, and the 100 highest-expressed genes in aortas of high-fat, high-cholesterol fed New Zealand White (NZW) rabbits (data from [94]), and illustrates similarities and differences between these two translational models of atherosclerosis. In this example, the lists of the highest-expressed genes (and thus presumably most important “housekeeping” genes involved in the disease) are independently analyzed to identify the common overrepresented GO categories for each list (i.e., for both rabbit and mouse aortas). In addition, VLAD allows visual comparison between two model organisms, such as mice vs. rabbits (Figure 7D), to pinpoint the commonalities and differences in “themes” of gene expression changes, based on differences in the statistical *P*-value of the over-represented GO terms (i.e., the relative width of the bar, Figure 7D). For example, “angiogenesis” genes have “more significant” over-representation among the 100 highest-expressed genes in rabbit atherosclerotic aortas, and “phagocytosis, engulfment” genes have “more significant” over-representation in the mouse model. In the interactive VLAD version, the output also has lists of genes from the user’s input associated with each overrepresented GO term, *p*-values, FDR *q*-values, etc. In particular, the ability to upload own “universe set” of genes allows for more precise identification of over- and underrepresented ontologies, while the ability to upload any ontology from OBO Foundry allows for the exploration of additional ontologies such as Mammalian Phenotype (Figure 7B,C) [80]. Importantly, in the online version of VLAD, GO annotations, as well as nomenclature of mouse and human genes are automatically updated weekly [90], although local installation of VLAD requires the individual laboratory to manually update gene annotations from GO. Similar tools, such as AmiGO [78], BiNGO [93], DAVID [92], GOrilla [91] (Table 1), are also very popular free peer-reviewed public resources to identify GO term overrepresentations in the lists of genes; however, many of these otherwise excellent tools lag behind in updating their gene-to-ontology annotations and mappings by as much as three to four years. Similar idea of measuring and testing overrepresentation within a group of genes of interest is implemented in Gene Set Enrichment Analysis (GSEA) [86], and commercial platforms such as Ingenuity Pathway Analysis (IPA) [95] and Pathway Studio [96].

Another useful tool to identify specific pathways in the large-scale gene expression data is MetaCyc [87], which contains a collection of curated biochemical pathways, annotated with organism-specific data on genes, pathways, proteins and compounds. MetaCyc tool, Cellular Overview, allows the user to upload gene expression data and visualize the expression upon the entire metabolic map while simultaneously retaining the ability to focus on individual pathways affected by disease or condition, such as aorta samples from atherosclerosis samples [85]. For mammals, curated databases currently include human [97], mouse [98] and cattle [99]. Differentially expressed gene lists can also be overlaid onto existing cellular pathways using portals such as Reactome [89] or the Kyoto Encyclopedia of Genes and Genomes (KEGG) [88] to explore potential secondary pathways, and dysregulated pathways specific to atherosclerotic pathology or healthy samples. Importantly, research community involvement in the process of gene annotation and curation, including creation of disease-specific ontology terms, improves the precision and quality of these resources to atherosclerosis and heart disease research [79].

Precision medicine classifies individuals according to their underlying susceptibility, prognosis, or targeting potential treatment response. Transcriptomics is an exciting tool for precision medicine, as it allows for a quick, unbiased and cost-effective identification of potential specific targets based on their under- or overexpression in the individual disease, without the need for a much more complex patient whole-genome analysis. Classifying patients based on symptoms is limited because symptoms often arise from numerous origins or multimodal pathways, as is the case with atherosclerosis. Biomedical researchers in both clinical and basic research settings need to choose transcriptome analysis to the specific characteristics of disease, and its pathology, to detect changes in the target molecular, cellular and physiological pathways under scientific scrutiny. Transcriptomics is a robust method to measure both common and unique pathways simultaneously. For unbiased detection in molecular and cellular pathways, researchers need to use a variety of tools, from read alignment to ontological analysis. Indeed, analyzing the data produced by transcriptome analysis facilitates researchers to explore gene functions, expression levels, differential gene expression, organismal responses to environmental and developmental changes, etc. in their totality, rather than narrowly focusing on specific, however they may seem to be important, pathways or genes. Embracing unbiased approaches to gene expression analysis can allow for the identification of novel disease biomarkers or even highly specific drugs, particularly impressive in the field of cancer [100], but also adopted by cardiovascular disease research community, such as discovery of the role of the RNA editing gene *ADAR1* in atherosclerosis [101], or the upregulation of estrogen receptor signaling pathways in women with myocardial infraction with nonobstructive coronary artery disease caused by atherosclerosis [102]. The ongoing NHLBI TOPmed (Trans-omics for precision medicine) effort to generate ~150,000 individual genomes’ sequences and ~50,000 transcriptomes for the most prevalent cardiovascular diseases, including coronary artery disease induced by atherosclerosis, will undoubtedly lead to new exciting discoveries [103]. Thus, when analyzing transcriptomes of samples, the key focus is the difference of expression levels of various groups of functionally related genes. Although the application of transcriptomics to the field of atherosclerosis as precision medicine is in its infancy, with these few aforementioned examples [101,102], its usefulness is actively being tested [103]. This paper serves as a resource article for tools, especially for investigators with limited experience, to embrace transcriptomic techniques applicable in atherosclerosis research, and ultimately to fulfill their promise for precision medicine.

## 5. Conclusions

Transcriptome analysis is an exciting tool, whose efficacy and efficiency are continually improving. The variety of platforms available to perform such analyses is a great advantage to laboratories both large and small, and the high-throughputs for some of these technologies provide rapid results with great accuracy. Identification of affected pathways using transcriptomics bioinformatics tools will allow researchers and clinicians to make a focused and informed decision upon the genes to concentrate on as potential therapeutic targets in precision medicine. The application of transcriptomics can facilitate the exploration of underlying pathogenic mechanisms, identification of genetic variants, determination of treatment effects, including screening for molecular biomarkers. Importantly, expression signatures in diseased phenotypes may pinpoint precise interventions required to alleviate the disease state, a goal of precision medicine, without a need for the cost prohibitive personalized assembly and deep analysis of patient’s genome. Thus, transcriptomics can classify individuals while simultaneously facilitate discovery, testing, and the validation of new therapeutics for patients with atherosclerosis, defined at the cellular and molecular levels.

## Figures and Tables

**Table 1:** Biomedical Ontology and Pathway Databases and Ontology/Pathway Enrichment Tools.

Resource	Description	URL	Ref
**Databases:**			
Gene Ontology	Central repository of terms describing gene functions across multiple biological systems	http://geneontology.org/	[77]
Mammalian Phenotype Ontology	Biomedical curators’ and community database of ontological terms for annotating phenotypic data	http://www.informatics.jax.org/vocab/mp_ontology/	[80]
Human Disease Ontology	Ontology for human disease cross-mapped to MeSH, ICD, NCI’s thesaurus, SNOMED and OMIM.	http://disease-ontology.org/	[81]
Protein Ontology	Ontology of protein-related entities, their explicit definitions, and relationships between them.	https://proconsortium.org/pro/pro.shtml	[84]
Open Biological Ontologies	Collaborative effort to specify and implement best principles and practices in ontology development. Contains links to all Ontologies.	http://obofoundry.org/	[85]
MSigDB	A collection of annotated gene sets, such as canonical pathways gene sets, for use with GSEA.	http://software.broadinstitute.org/gsea/msigdb/index.jsp	[86]
MetaCyc	A curated database of experimentally elucidated metabolic pathways for many organisms.	https://metacyc.org/	[87]
KEGG	A collection of maps representing metabolism, pathways, and associated genes.	https://www.genome.jp/kegg/	[88]
Reactome	A free, open-source, curated and peer-reviewed pathway database.	https://reactome.org/	[89]
**Tools:**			
VLAD	Tool for identification of statistically significant over- or under-represented ontology terms in lists of genes. GO gene – function annotations for human and mouse, and MP gene – phenotype annotations for mouse are pre-loaded. Allows uploading user-specified ontologies and gene – ontology mappings. Updated weekly.	http://proto.informatics.jax.org/prototypes/vlad/	[90]
AmiGO	Allows users to query, browse and visualize ontologies and gene annotation data for many species. Updated weekly.	http://amigo.geneontology.org/amigo	[78]
GOrilla	A tool to identify and visualize enriched GO terms in gene lists. Can either search for GO terms at the top of a ranked gene list, or compare a target gene list to a background gene list.	http://cbl-gorilla.cs.technion.ac.il/	[91]
DAVID	A set of tools to identify overrepresented features in large lists of genes.	https://david.ncifcrf.gov/	[92]
BinGO	Cytoscape tool to visualize statistically overrepresented GO terms in a list of genes.	http://apps.cytoscape.org/apps/bingo ^1^	[93]
GSEA	A tool to determine if a gene set has significant differences between two biological states.	http://software.broadinstitute.org/gsea/downloads.jsp ^1^	[86]

^1^ Download link for a stand-alone tool.
